# The association between metabolic dysfunction-associated steatotic liver disease, cardiovascular and cerebrovascular diseases and the thickness of carotid plaque

**DOI:** 10.1186/s12872-023-03580-6

**Published:** 2023-11-11

**Authors:** Yunqian Huang, Yuqun Wang, Zhengguang Xiao, Shengqi Yao, Yuhua Tang, Linjun Zhou, Qin Wang, Yanchun Xie, Lixia Zhang, Yan Zhou, Ying Lu, Wenqian Zhu, Man Chen

**Affiliations:** 1 Department of Ultrasound Medicine, Tongren Hospital, Shanghai Jiao Tong University School of Medicine, Shanghai, China; 2 Department of Radiology, Tongren Hospital, Shanghai Jiao Tong University School of Medicine, Shanghai, China; 3 Department of Neurology, Tongren Hospital, Shanghai Jiao Tong University School of Medicine, Shanghai, China

**Keywords:** Metabolic dysfunction-associated steatotic liver disease, Cardiovascular disease, Cerebrovascular disease, Carotid plaque, Free fatty acid, Ultrasound

## Abstract

**Background:**

The relationship between metabolic dysfunction-associated steatotic liver disease (MASLD) and atherosclerosis has been controversial, which has become a hit of recent research. The study aimed to explore the association between MASLD, cardiovascular and cerebrovascular diseases (CCVD), and the thickness of carotid plaque which was assessed by ultrasound.

**Methods:**

From September 2018 to June 2019, 3543 patients were enrolled. We asked participants to complete questionnaires to obtain information. All patients underwent liver ultrasound and bilateral carotid ultrasound to obtain carotid intima-media thickness (IMT) and maximum carotid plaque thickness (CPT). Hepatic steatosis was quantified during examination according to Hamaguchi’s ultrasonographic score, from 0 to 6 points. A score < 2 was defined as without fatty liver, and a score ≥ 2 was defined as fatty liver. Information about blood lipids was collected based on the medical records.

**Results:**

We found common risk factors for CCVD events, MASLD, and atherosclerosis. There was a significant correlation between MASLD and carotid plaque, but not with CPT. No association was found between MASLD and CCVD events. CPT and IMT were thicker in CCVD patients than in non-CCVD patients. No significant difference was found between IMT and CPT in MASLD patients and non-MASLD patients. CCVD was independently and consistently associated with higher IMT, and free fatty acid (FFA).

**Conclusions:**

According to our results, we recommend carotid ultrasound examination of the patients when FFA is increased, regardless of the presence of risk factors and MASLD. Due to the distribution of CPT of both CCVD and MASLD patients in the CPT 2-4 mm group, contrast-enhanced ultrasound is necessary to assess the vulnerability of the plaque when CPT ≥ 2 mm. Timely treatment of vulnerable plaques may reduce the incidence of future CCVD events.

## Background

Overweight is a global epidemic, affecting an increasing number of the world population [1]. In China, the average body mass index (BMI) and overweight rate among adults have been rising steadily since the early 1980s [2]. Approximately 30% of the population in the Western world suffer from metabolic dysfunction-associated steatotic liver disease (MASLD), as the incidence rate of overweight, diabetes mellitus (DM), and metabolic syndrome continue to rise [3, 4]. A study from The Lancet showed that the prevalence of fatty liver in the Chinese population was 21.2% [5]. Shanghai is one of the fastest-growing cities in China. It is the city with the longest life expectancy among all cities in China and has made a great contribution to the human development index (HDI) of China [6]. Due to the affluent life, the fast pace of work, and the unhealthy lifestyle of permanent residents in Shanghai, there are a large number of residents with fatty liver disease.

The most common cause of death in patients with MASLD is cardiovascular and cerebrovascular diseases (CCVD) events. CCVD has high morbidity, high disability rate, and high mortality rate. This has become grown up to be a global public health and livelihood issue. Several previous studies [7–12] have shown that plasma lipids were an important mediator between MASLD and CCVD risk, that liver fat content was associated with increased carotid intima-media thickness (IMT), and that there was a need for routine screening of the carotid arteries in patients with fatty liver. Patients with MASLD have a significantly increased coronary atherosclerotic burden, and appropriate treatment of MASLD is needed to reduce future cardiac events [13]. However, most of these studies have mostly focused on the relationship between carotid intima-media thickness, coronary arteries, and MASLD, with fewer studies on the relationship between MASLD and carotid plaque thickness. The relationship between MASLD and atherosclerosis has been controversial, which has become a hit of recent research. Systemic inflammation, endothelial dysfunction, hepatic insulin resistance, increased oxidative stress and altered lipid metabolism are key factors linking MASLD and CCVD risk mechanisms. The contribution of MASLD to the growth of plaque thickness after atherosclerotic plaque formation is unclear.

Accordingly, the purpose of this study was to explore the association between MASLD, CCVD, and the thickness of carotid plaque which was assessed by ultrasound.

## Methods

### Patient population

**Fig. 1:**
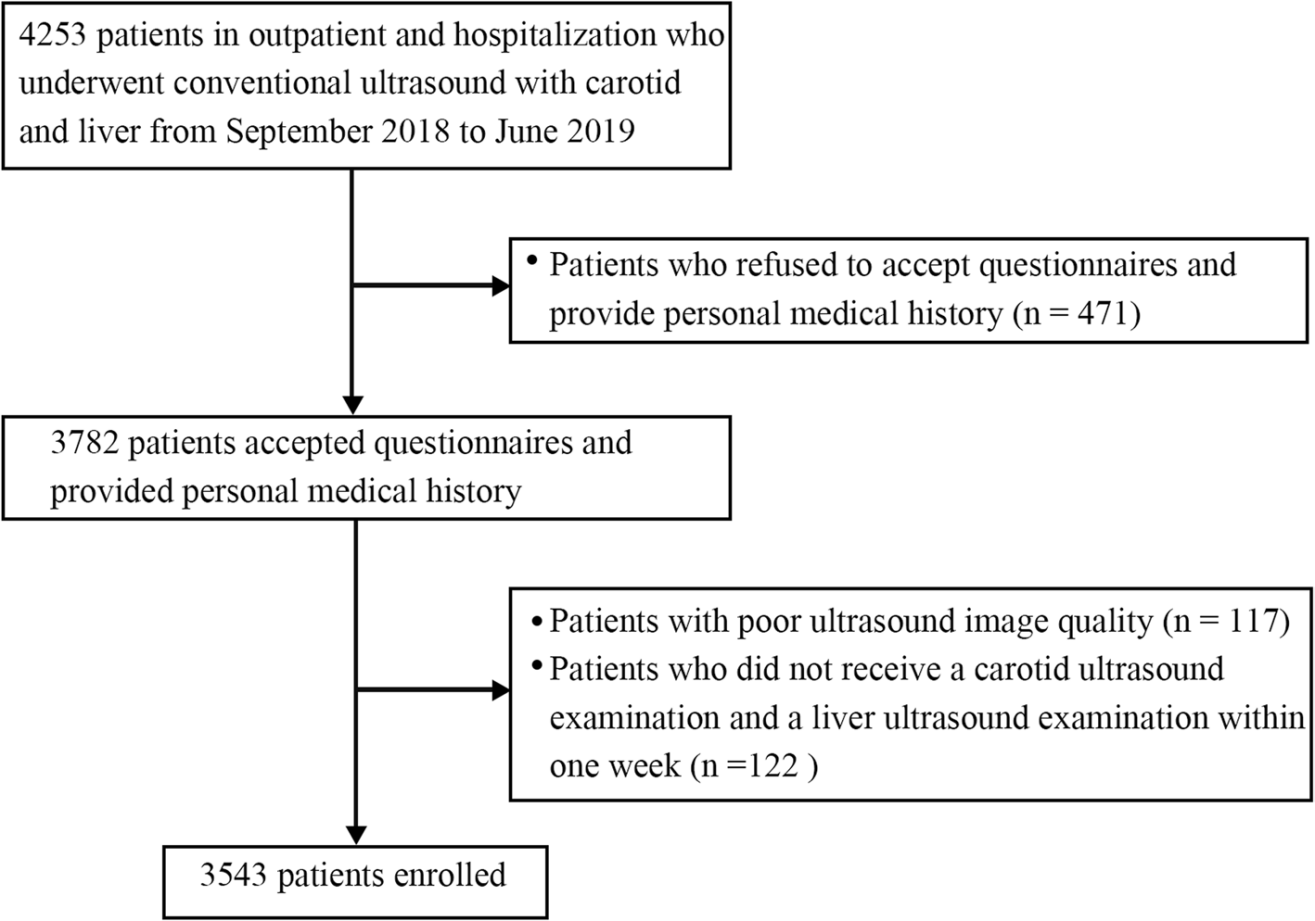
Patient selection

We retrospectively identified 4253 patients in outpatient and hospitalization who underwent conventional ultrasound with carotid and liver in our hospital from September 2018 to June 2019. The study protocol was subject to approval by the Institutional Ethics Review Committee (Shanghai Tongren Hospital 2018–030) and written informed consent was issued from all participants. Seven hundred and ten patients were excluded with exclusion criteria including (a) patients who refused to accept questionnaires and provide personal medical history (*n* = 471), (b) patients with poor ultrasound image quality (*n* = 117), and (c) patients who did not receive a carotid ultrasound examination and a liver ultrasound examination within one week (*n* = 122). Finally, 3543 patients were enrolled (Fig. 1).

### Carotid ultrasound imaging

All patients underwent bilateral duplex ultrasound evaluation of the carotid arteries. To identify atherosclerotic lesions and for assessment of plaque thickness, transverse and longitudinal sweeps were recorded from the proximal common carotid artery, just above the clavicle, to the common carotid artery bifurcation and then following the internal carotid artery as far as possible, at an angle perpendicular to the neck. The database with carotid ultrasound images was collected by an ultrasound physician (YQH, with 13 years of experience with ultrasound), who was blinded to participant history, using a 6–10 MHz linear probe (9L4; SIEMENS ACUSON OXANA2). The image parameters were set to show the plaque and surrounding tissue as clearly as possible. Before the formal study, the ultrasound physician selected 100 images for review.

**Fig. 2:**
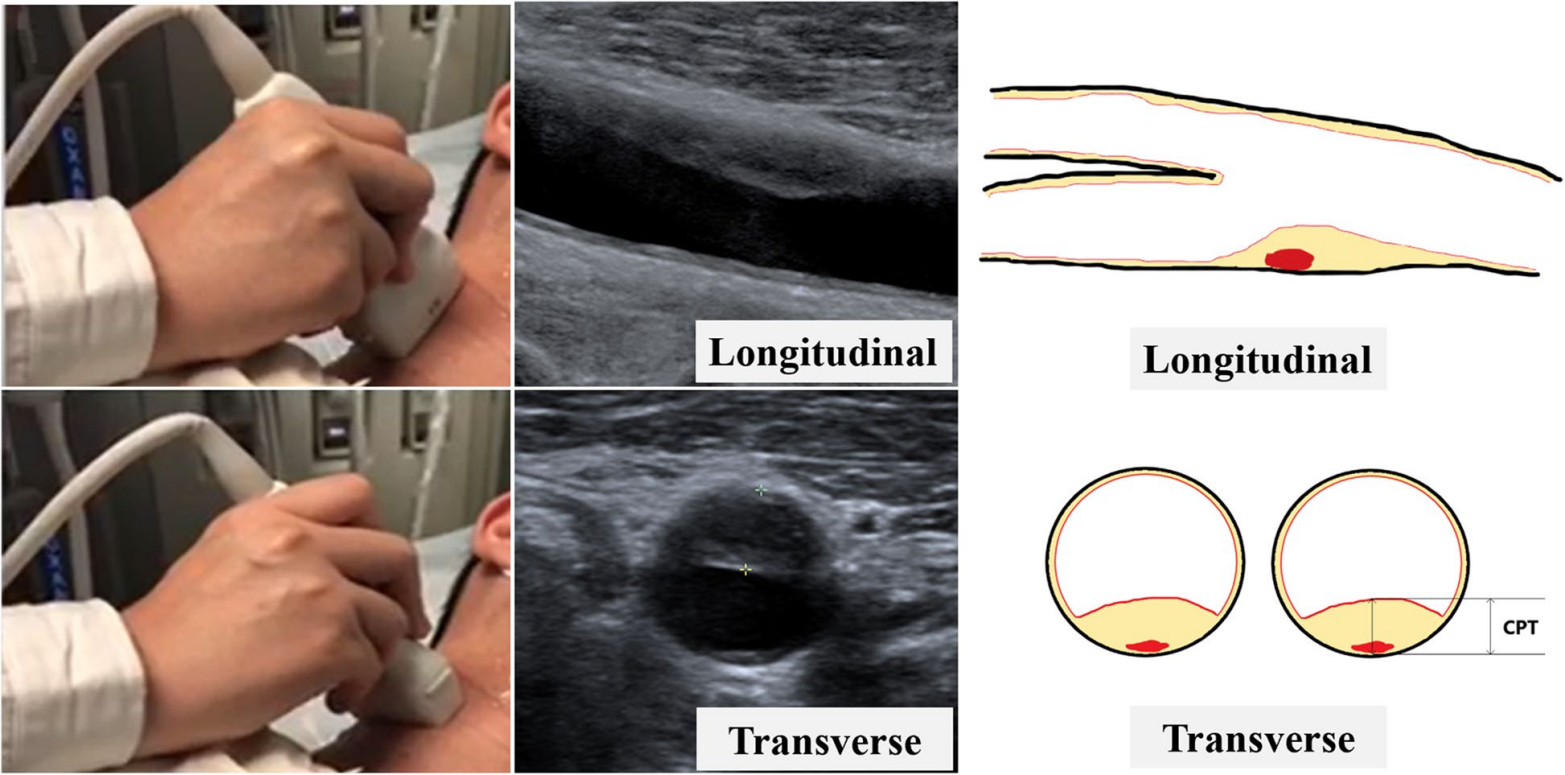
Carotid ultrasound imaging and carotid plaque thickness (CPT) measurement

All patients were examined in a supine position. The carotid artery was scanned from the clavicle, upwards to the bifurcation of the carotid artery, and finally towards the internal and external carotid arteries. Each segment of carotid artery was examined and the thickest IMT was recorded [14]. The IMT was defined as the thickness of the inner two layers of the carotid artery: the intima and the media. Carotid plaque (CP) was defined as a focal region with a thickness > 1.5 mm as measured from the media adventitia interface to the lumen-intima interface or as the presence of focal wall thickening (at least 50% greater than that of the surrounding vessel wall) [15]. If a plaque was identified, the view showing the thickest cross-section of the plaque from either side (right and left carotid artery) was used to measure the maximal carotid plaque thickness with electronic calipers. CPT was measured by finding the thickest part of the plaque in the cross-sectional and then measuring the radial distance from the media–adventitia interface to the intima–lumen interface towards the center of the arterial lumen [16, 17]. In patients with multiple plaques, only the thickest one was observed and recorded for analysis **(**Fig. 2**)**. After completing the first IMT and CPT measurement, the measurement was repeated one week later. And the consistency was obtained before proceeding to the next step.

### MASLD ultrasound imaging

**Fig. 3:**
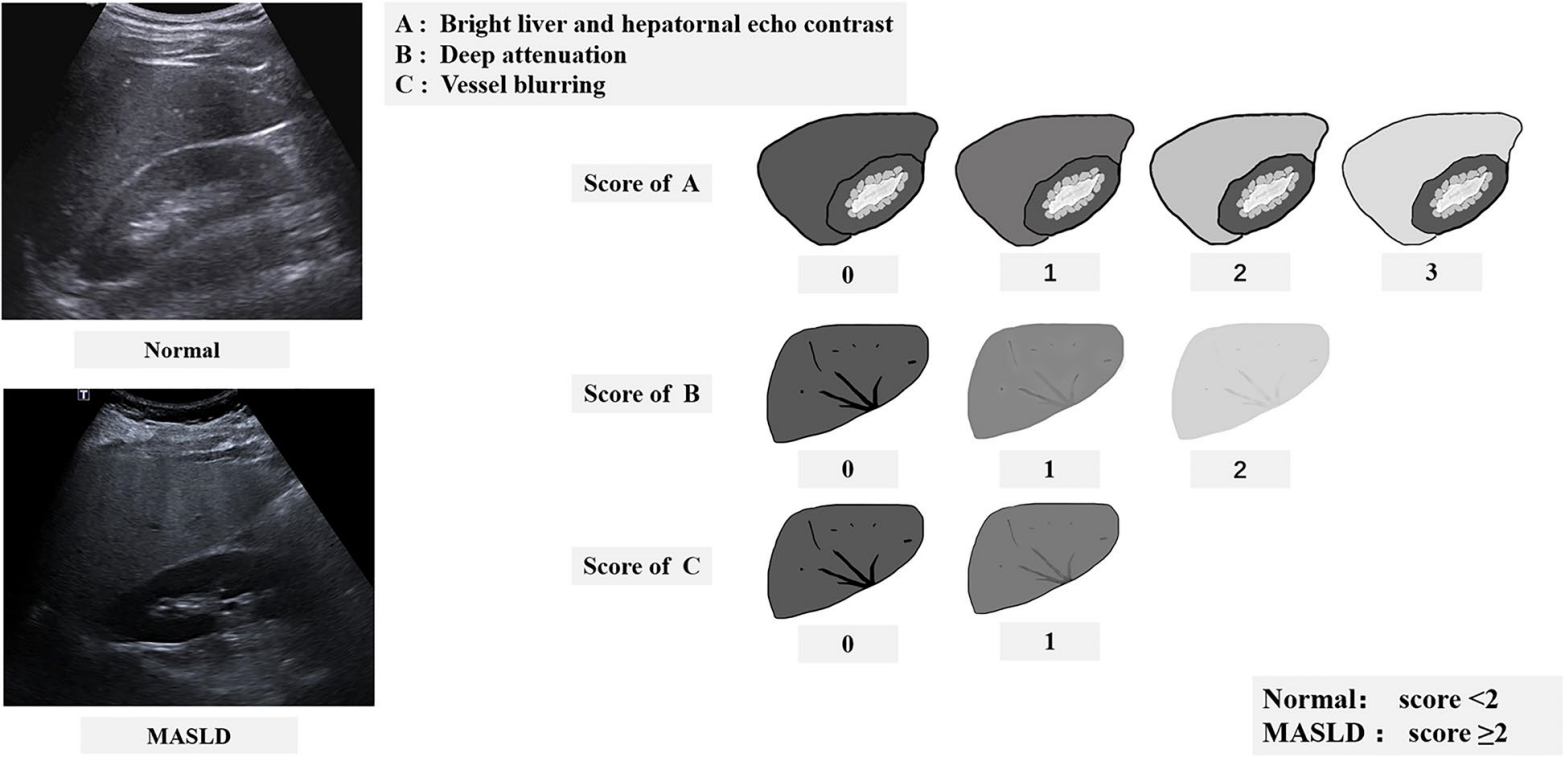
Metabolic dysfunction-associated steatotic liver disease (MASLD) ultrasound imaging and MASLD assessment

All patients underwent liver ultrasound examination when they underwent carotid ultrasound in 1 week without excessive alcohol abuse (weekly alcohol consumption ≤ 210 g in men and ≤ 140 g in women) and other liver diseases. The subjects were all in the supine position, scanning the liver under the ribs and between the ribs to observe its size and shape, the echo of the liver parenchyma, and whether the blood vessels in the liver were positive. At the same time, the echoes of the liver and right kidney parenchyma were compared. Hepatic steatosis was quantified during examination according to Hamaguchi’s ultrasonographic score [18, 19], from 0 to 6 points, based on the hepatorenal contrast, bright hepatic echoes, deep attenuation, and vessel blurring. A score < 2 was defined as without fatty liver, and a score ≥ 2 was defined as fatty liver (Fig. 3).

The database with liver ultrasound images was collected by three ultrasound physicians with at least five years of experience in abdomen ultrasound. We randomly selected 200 liver images from the database. These images were sent respectively to the three ultrasound physicians, who were blinded to the clinical information of patients. These images were evaluated according to the standard of Hamaguchi’s ultrasonographic score by physicians A, B, and C. The consistency of the 200 images assessed by three physicians was evaluated. A satisfactory agreement was obtained before the formal collection of patient images began.

### Baseline examination

Trained investigators interviewed participants face-to-face, using a standard questionnaire to obtain their demographics, lifestyle, disease history, and the potential risk factors related to CCVD.

Smoke (Regular consumption of cigarettes in the past 6 months was defined as a current smoker).

Alcohol consumption (Excessive alcohol consumption was defined as > 210 g per week for men and > 140 g per week for women).

Hypertension (Patients who are taking hypertensive drugs are also included).

Diabetes (Patients who are taking glucose-lowering drugs or insulin therapy are also included etc.)

Dyslipidemia (including triglycerides ≥ 2 mmol/L, high-density lipoprotein cholesterol ≥ 0.7 mmol/L, low-density lipoprotein cholesterol ≥ 3.4 mmol/L, etc.) [20].

Exercise status (Lack of exercise was defined as exercising less than three times a week for less than half an hour each time).

Obstructive sleep apnea hypoventilation (Previously diagnosed).

History of atrial fibrillation (AF) (Previously diagnosed as AF).

Family history of CCVD (The presence of ischemic heart disease, myocardial infarction, ischemic and hemorrhagic stroke, transient ischemic attack, and peripheral arterial disease within three generations of the patient's immediate family was defined as the presence of a family history of CCVD).

Physical examination was performed before the ultrasound examination, including the measurements of BMI and blood pressure. Participants' body weight and height were obtained in light clothes and bare feet to the nearest 0.1 kg and 0.1 cm, respectively. BMI was calculated as the weight (kg) divided by the square of the height (m). Overweight was defined as BMI ≥ 26 [21, 22]. Blood pressure was measured on the non-dominant arm in a seated position by an automated electronic sphygmomanometer three times consecutively at 1 min intervals, after at least 10 min rest.

Hypertension and DM were determined according to the 2013 Hypertension Clinical Practice Guidelines [23] and the 2017 Standards of Medical Care in Diabetes [24], respectively.

The medical records of all subjects were reviewed for information about blood lipids and CCVD events. CCVD events were defined as stroke, transient ischemic attack, stroke death, and myocardial infarction. Stroke was defined as a neurological deficit lasting at least 24 h or until death and accompanied by a brain imaging finding associated with stroke. A transient ischemic attack was defined as a neurological deficit lasting between the 30 s and 24 h but not accompanied by a brain imaging finding associated with stroke [25]. Myocardial infarction was defined according to the American College of Cardiology and American Heart Association Guidelines [26]. Information about blood lipids [fasting blood glucose (FBG), lipid profiles total cholesterol (TC), triglycerides (TG), high-density lipoprotein cholesterol (HDL-C), low-density lipoprotein cholesterol (LDL-C), lipoprotein(a), apolipoprotein A (APOA), apolipoprotein B (APOB), apolipoprotein E (APOE), free fatty acid (FFA), small and dense low-density lipoprotein cholesterol (SDLDL-C), non-high-density lipoprotein cholesterol (NHDL-C)] was collected based on the medical records.

### Statistical analysis

Continuous data were presented as mean ± standard deviation or as median.

(interquartile range), as appropriate (skewed distribution). Continuous normally distributed variables were compared using the student’s test for independent samples and analysis of variance. Alternatively, the Mann–Whitney U test was used for independent samples and the Wilcoxon test for repeated measurements. The proportion of categorical variables was compared using a Chi-square test or the Fisher exact test. *P* < 0.05 was considered to indicate statistical significance.

### Inter-observer and intra-observer agreement in MASLD and CPT assessment

The consistency assessment of the three ultrasound physicians on the diagnosis of MASLD was completed by Kendall's W test and the kappa test. The consistency of carotid plaque measurement was completed by intra-class correlation (ICC) estimates. Interobserver variations were investigated by using the Kappa statistics. ICC estimates and their 95% confidence intervals were calculated based on a mean-rating, absolute-agreement, 2-way mixed-effects model.

Strong agreement was observed for the inter-observer assessment of MASLD by three ultrasound physicians (A, B, and C), the Kendall's W test was 0.950 (Physician A versus B [k = 0.918, 95% CI 0.858–0.969], Physician B versus C [k = 0.908, 95% CI 0.843–0.959], Physician C versus A [k = 0.907, 95% CI 0.855–0.960]), indicating good reliability and rated as "mostly perfect". For the intra-observer reproducibility of CPT and IMT, the ICC value was 0.994 (95% CI 0.991–0.996) and 0.937(95% CI 0.917–0.953), Kendall's W test was 0.996 and 0.970, indicating excellent reliability and rated as "mostly perfect" (Table 1).

**Table 1:** Inter-observer agreement in the assessment of MASLD by three ultrasound physicians, and Intra-observer agreement of CPT by ultrasound physician at two different times

**Inter-observer agreement in assessment of MASLD by three ultrasound physicians (A, B, C)**					
	**A&B**	**B&C**	**A&C**	**Kendall's W**	**Agreement**
**Kappa**	0.918	0.908	0.907	0.950	**Almost perfect**
***P-*value**	0.000	0.000	0.000		
**95% CI**	0.858-0.969	0.843-0.959	0.855-0.960		
**Intra-observer Agreement of CPT by ultrasound physician at two different times**					
**CPT**	**ICC**	**Kendall's W**	**P-value**	**95% CI**	**Agreement**
	0.994	0.996	0.000	0.991-0.996	**Almost perfect**
**IMT**	0.937	0.970	0.000	0.917-0.953	**Almost perfect**

*MASLD* Non-alcoholic fatty liver disease, *CPT* Carotid plaque thickness, *ICC* Intra-class correlation, *IMT* Intima-media thickness, *CI* Confidence interval

## Results

### The characteristics of the baseline

Study participants included 3543 men and women, aged from 20 to 88 years old. The mean age of patients was 64.12 ± 11.47 years, and 45.1% of them were male. Overall, 26.3% of the patients were overweight, 16.7% of the patients were current smokers, 57.4% of the patients had hypertension, 21.1% of the patients had DM, 25.9% of the patients had a family history of CCVD, 12.6% of the patients had a personal history of CCVD, 45.1% of the patients had dyslipidemia, 76.9% of the patients were lack of exercise, and 4.2% of the patients had obstructive sleep apnea. The potential risk factors associated with MASLD, CP, and CCVD were shown in Table 2. No significant association was found between a family history of CCVD, dyslipidemia and CCVD events. In our study, age, BMI, gender, smoking, hypertension, DM, personal history of CCVD, history of AF, lack of exercise, fatty liver, obstructive sleep apnea, and CP were identified as potential risk factors for CCVD (*P* < 0.05). CPs were present in 60.5% of MASLD patients. Compared with those with non-MASLD, subjects with MASLD were younger. Participants with MASLD had a higher elevated blood lipid level and a higher BMI in comparison with those with non-MASLD. There seemed to be no association between fatty liver disease and cardio-cerebrovascular events.

**Table 2:** Potential risk factors and lipids associated with MASLD, CP, and CCVD in all patients (*N* = 3543)

		**All (3543)**	**Non- MASLD (2194)**	**MASLD (1349)**	***P*-value**	**Non- Carotid Plaque (1491)**	**Carotid Plaque (2052)**	***P*-value**	**Non- CCVD (3098)**	**CCVD (445)**	***P*-value**
**Age, y (mean ± SD)**		3543	64.54 ± 12.016	63.45 ± 10.391	**0.006**	63.63 ± 12.146	65.30 ± 9.531	**0.000**	63.28 ± 11.204	69.99 ± 11.552	**0.000**
**BMI**		3543	23.37 ± 4.875	25.34 ± 3.365	**0.000**	23.60 ± 4.748	25.36 ± 3.397	**0.000**	24.05 ± 3.439	24.64 ± 8.735	**0.009**
**Gender**	**F**	1945 (54.9%)	1260 (57.4%)	685 (50.8%)	**0.000**	946 (63.4%)	999 (48.7%)	**0.000**	1772 (57.2%)	173 (38.9%)	**0.000**
	**M**	1598 (45.1%)	934 (42.6%)	664 (49.2%)		545 (36.6%)	1053 (51.3%)		1326 (42.8%)	272 (61.1%)	
**Overweight**	**No**	2611 (73.7%)	1796 (81.9%)	815 (60.4%)	**0.000**	1093 (73.3%)	1518 (74.0%)	0.341	2296 (74.1%)	315 (70.8%)	0.150
	**Yes**	932 (26.3%)	398 (18.1%)	534 (39.6%)		398 (26.7%)	534 (26.0%)		802 (25.9%)	130 (29.2%)	
**Smoke**	**No**	2951 (83.3%)	1884 (85.9%)	1067 (79.1%)	**0.000**	1308 (87.7%)	1643 (80.1%)	**0.000**	2607 (84.2%)	344 (77.3%)	**0.001**
	**Yes**	592 (16.7%)	310 (14.1%)	282 (20.9%)		183 (12.3%)	409 (19.9%)		491 (15.8%)	101 (22.7%)	
**Hypertension**	**No**	1508 (42.6%)	1068 (48.7%)	440 (32.6%)	**0.000**	822 (55.1%)	686 (33.4%)	**0.000**	1396 (45.1%)	112 (25.2%)	**0.000**
	**Yes**	2035 (57.4%)	1126 (51.3%)	909 (67.4%)		669 (44.9%)	1366 (66.6%)		1702 (54.9%)	333 (74.8%)	
**DM**	**No**	2794 (78.9%)	1806 (82.3%)	988 (73.2%)	**0.000**	1303 (87.4%)	1491 (72.7%)	**0.000**	2521 (81.4%)	273 (61.3%)	**0.000**
	**Yes**	749 (21.1%)	388 (17.7%)	361 (26.8%)		188 (12.6%)	561 (27.3%)		577 (18.6%)	172 (38.7%)	
**Family history of CCVD**	**No**	2625 (74.1%)	1654 (75.4%)	971 (72.0%)	**0.027**	1082 (72.6%)	1543 (75.2%)	0.081	2300 (74.2%)	325 (73.0%)	0.603
	**Yes**	918 (25.9%)	540 (24.6%)	378 (28.0%)		409 (27.4%)	509 (24.8%)		798 (25.8%)	120 (27.0%)	
**History of AF**	**No**	3259 (92.0%)	2029 (92.5%)	1230 (91.2%)	0.181	1420 (95.2%)	1839 (89.6%)	**0.000**	2881 (93.0%)	378 (84.9%)	**0.000**
	**Yes**	284 (8.0%)	165 (7.5%)	119 (8.8%)		71 (4.8%)	213 (10.4%)		217 (7.0%)	67 (15.1%)	
**Dyslipidemia**	**No**	1945 (54.9%)	1531 (69.8%)	414 (30.7%)	**0.000**	859 (57.6%)	1086 (52.9%)	**0.006**	1695 (54.7%)	250 (56.2%)	0.575
	**Yes**	1598 (45.1%)	663 (30.2%)	935 (69.3%)		632 (42.4%)	966 (47.1%)		1403 (45.3%)	195 (43.8%)	
**Lack of exercise**	**No**	818 (23.1%)	521 (23.7%)	297 (22.0%)	0.250	375 (25.2%)	443 (21.6%)	**0.014**	754 (24.3%)	64 (14.4%)	**0.000**
	**Yes**	2725 (76.9%)	1673 (76.3%)	1052 (78.0%)		1116 (74.8%)	1609 (78.4%)		2344 (75.7%)	381 (85.6%)	
**Obstructive sleep apnea**	**No**	3393 (95.8%)	2132 (97.2%)	1261 (93.5%)	**0.000**	1434 (96.2%)	1959 (95.5%)	0.312	2972 (95.9%)	421 (94.6%)	0.207
	**Yes**	150 (4.2%)	62 (2.8%)	88 (6.5%)		57 (3.8%)	93 (4.5%)		126 (4.1%)	24 (5.4%)	
**CP**	**No**	1491 (42.1%)	958 (43.7%)	533 (39.5%)	**0.016**	/	/	/	1398 (45.1%)	93 (20.9%)	**0.000**
	**Yes**	2052 (57.9%)	1236 (56.3%)	816 (60.5%)		/	/	/	1700 (54.9%)	352 (79.1%)	
**CCVD**	**No**	3098 (87.4%)	1931 (88.0%)	1167 (86.5%)	0.192	1398 (93.8%)	1700 (82.8%)	**0.000**	/	/	/
	**Yes**	445 (12.6%)	263 (12.0%)	182 (13.5%)		93 (6.2%)	352 (17.2%)		/	/	/
**Glucose** (mmol/L)		3543	4.887 ± 3.390	5.324 ± 3.279	0.163	4.691 ± 3.366	6.049 ± 3.107	**0.000**	4.691 ± 3.366	6.049 ± 3.107	**0.000**
**TC** (mmol/L)		3543	4.360 ± 1.046	4.502 ± 1.156	0.173	4.495 ± 1.072	4.210 ± 1.124	**0.001**	4.495 ± 1.072	4.209 ± 1.124	**0.011**
**HDL-C** (mmol/L)		3543	1.310 ± 0.395	1.173 ± 0.391	**0.000**	1.289 ± 0.416	1.168 ± 0.335	**0.001**	1.288 ± 0.415	1.168 ± 0.334	**0.003**
**TG** (mmol/L)		3543	1.252 ± 0.622	1.928 ± 1.574	**0.000**	1.555 ± 1.244	1.432 ± 0.855	0.300	1.555 ± 1.243	1.432 ± 0.854	0.300
**LDL-C** (mmol/L)		3543	2.779 ± 1.812	2.757 ± 0.985	0.875	2.746 ± 0.982	2.834 ± 2.472	0.695	2.746 ± 0.982	2.833 ± 2.471	0.695
**Lp(a)** (mg/L)		3543	230.1 ± 238.6	253.5 ± 278.9	0.346	237.9 ± 262.8	243.4 ± 235.1	0.833	237.8 ± 262.7	243.4 ± 235.0	0.833
**APOA** (g/L)		3543	1.364 ± 0.770	1.364 ± 0.962	1.000	1.373 ± 0.738	1.342 ± 1.098	0.725	1.372 ± 0.737	1.341 ± 1.098	0.725
**APOB** (g/L)		3543	0.849 ± 0.256	0.908 ± 0.255	**0.014**	0.875 ± 0.254	0.864 ± 0.263	0.662	0.875 ± 0.254	0.863 ± 0.263	0.662
**APOE** (g/L)		3543	3.911 ± 1.225	4.215 ± 4.214	**0.015**	4.067 ± 1.365	3.936 ± 1.255	0.343	4.067 ± 1.365	3.936 ± 1.254	0.343
**FFA** (mmol/L)		3543	0.542 ± 0.246	0.554 ± 0.203	0.596	0.219 ± 0.012	0.245 ± 0.022	**0.000**	0.522 ± 0.218	0.610 ± 0.245	**0.000**
**NHDL-C** (mmol/L)		3543	3.035 ± 0.995	3.337 ± 1.041	**0.002**	1.005 ± 0.054	1.065 ± 0.093	0.156	3.196 ± 1.005	3.046 ± 1.065	0.156
**SDLDL-C** (mmol/L)		3543	0.647 ± 0.376	0.842 ± 0.408	**0.000**	0.408 ± 0.022	0.379 ± 0.033	0.182	0.740 ± 0.407	0.685 ± 0.379	0.529

*MASLD* Non-alcoholic fatty liver disease, *CP* Carotid plaque, *CCVD* c Cardiovascular and cerebrovascular diseases, *BMI* Body mass index, *DM* Diabetes mellitus, *AF* Atrial fibrillation, *CP* Carotid plaque, *TC* Total cholesterol, *HDL-C*, High-density lipoprotein cholesterol, *TG* Triglycerides, *LDL-C* Low-density lipoprotein cholesterol, *Lp(a)* lipoprotein(a), *APOA* Apolipoprotein A, *APOB* Apolipoprotein B, *APOE* Apolipoprotein E, *FFA* Free fatty acid, *NHDL-C* Non-high-density lipoprotein cholesterol, *SDLDL-C* Small and dense low-density lipoprotein cholesterol, *SD* Standard deviation

### Distribution of IMT and CPT in the MASLD and CCVD groups among patients with carotid plaque

**Fig. 4:**
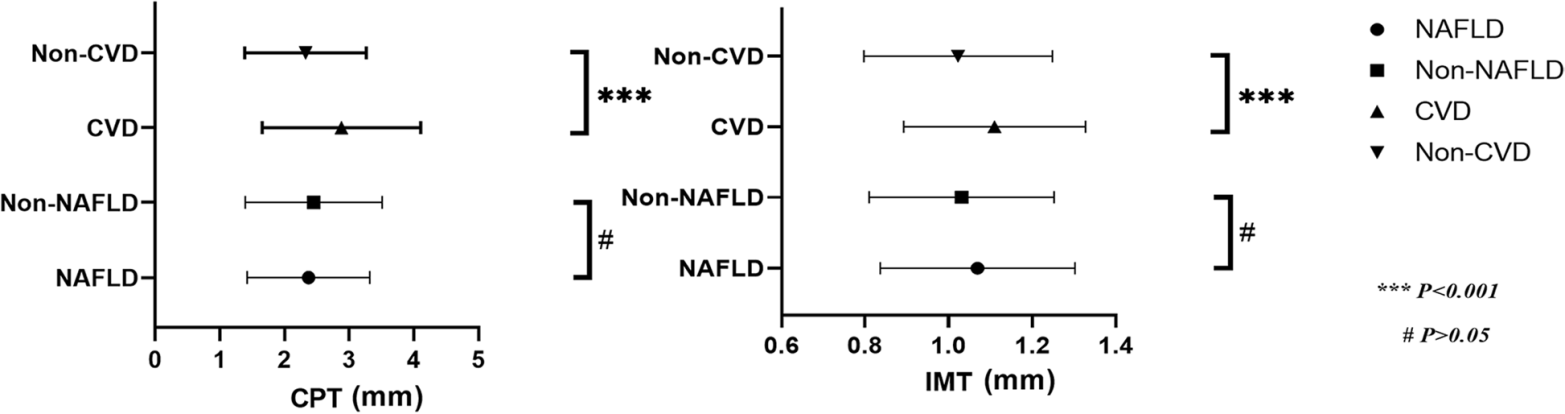
Distribution of intima-media thickness (IMT) and carotid plaque thickness (CPT) in the metabolic dysfunction-associated steatotic liver disease (MASLD) and cardiovascular and cerebrovascular diseases (CCVD) groups among patients with carotid plaque

There were 2052 patients with CP in our study cohort. Among them, 352 patients suffered from CCVD. The mean CPT with non-CCVD patients was 2.328 ± 0.935 mm, and with CCVD patients was 2.887 ± 1.214 mm (*P* = *0.000*). The mean IMT with non-CCVD patients was 1.022 ± 0.225 mm, and with CCVD patients was 1.110 ± 0.217 mm (*P* = *0.000*). 816 patients suffered from MASLD among 2052 patients with CP. The mean CPT with non-MASLD patients was 2.458 ± 1.051 mm, and with MASLD patients was 2.374 ± 0.945 mm (*P* > *0.05*). The mean IMT with non-MASLD patients was 1.031 ± 0.221 mm, and with MASLD patients was 1.069 ± 0.234 mm (*P* > *0.05*). CPT and IMT had no significant correlation between non-MASLD and MASLD **(**Fig. 4**)**. Participants with CCVD had a thicker CP thickness in comparison with those with non-CCVD. In both CCVD and MASLD patient groups, carotid plaque thickness was mostly distributed in the CPT 2-4 mm group.

### Multivariate logistic regression analysis of potential risk factors and lipids in CCVD events

**Fig. 5:**
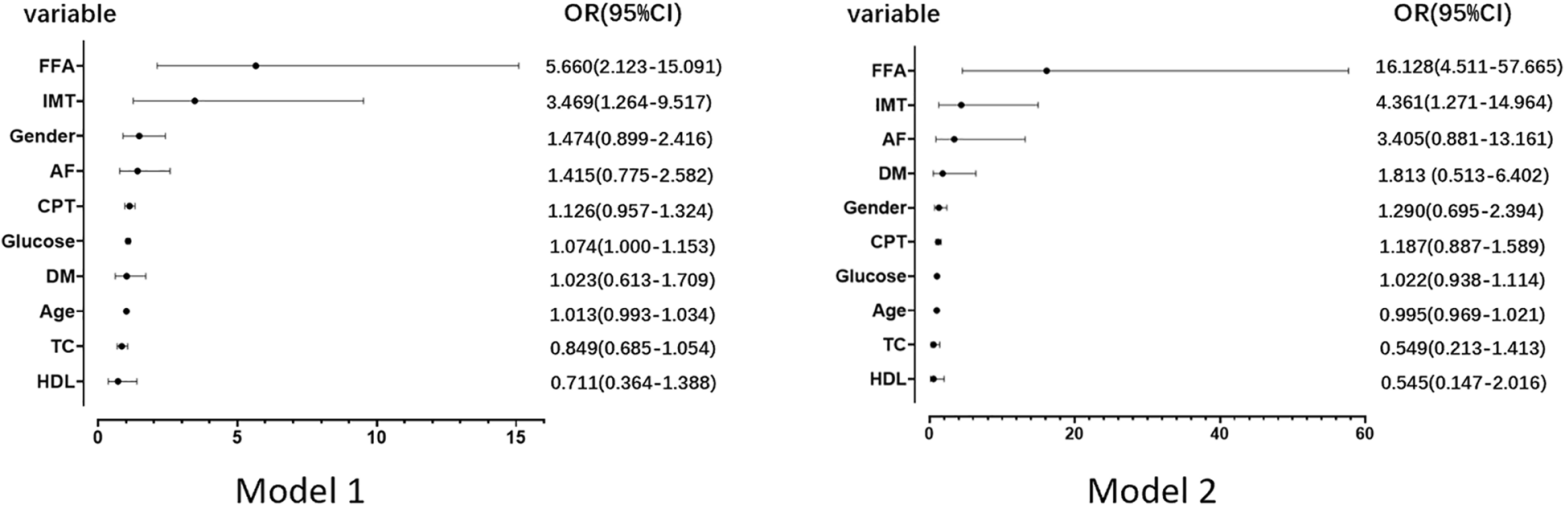
Forest plot representation with the multivariate logistic regression analysis of potential risk factors and lipids in cardiovascular and cerebrovascular diseases (CCVD) subjects. Model 1: unadjusted; Model 2: all potential risk factors including age, BMI, gender, smoking, hypertension, DM, personal history of cardiovascular and cerebrovascular diseases, history of AF, lack of exercise, fatty liver, obstructive sleep apnoea, and CP were adjusted. *CCVD* Cardiovascular and cerebrovascular diseases, *OR* Odds ratio, *CI* confidence interval, *DM* Diabetes mellitus, *AF* Atrial fibrillation, *IMT* Intima-media thickness, *CPT* Carotid plaque thickness, *TC* Total cholesterol, *HDL-C*, High-density lipoprotein cholesterol, *FFA* Free fatty acid

The univariate logistic regression analysis revealed a significantly increased risk of major access site complications for Male in gender(OR[odds ratio], 1.841; 95% CI, 1.217–2.785), age (OR: 1.027, 95% CI 1.009–1.045), DM (OR: 1.871, 95% CI 1.222–2.867), AF (OR: 1.788, 95% CI 1.027–3.113), IMT (OR: 5.756, 95% CI 2.287–14.489), CPT (OR:1.288, 95% CI 1.125–1.474), Glucose (OR: 1.127, 95% CI 1.060–1.198),TC (OR: 0.779, 95% CI 0.642–0.946), HDL (OR: 0.433, 95% CI 0.247–0.761)and FFA (OR: 5.161, 95% CI 2.123–12.541). These parameters were included in multivariate logistic regression analysis and the result was shown in Table 3. There seemed to be no association between fatty liver disease and cardio-cerebrovascular events. In the unadjusted Model 1, CCVD was independently and consistently associated with higher IMT and FFA. Regarding the CPT, statistically significant relationships with CCVD were observed less frequently and were less robust. After adjusting for all potential risk factors including age, BMI, gender, smoking, hypertension, DM, personal CCVD history, history of AF, lack of exercise, fatty liver, obstructive sleep apnoea, and CP, FFA was consistently associated independently with CCVD (OR: 16.128, 95% CI 4.511–57.665) and CPT was not significantly associated with CCVD (*P* = 0.249) **(**Fig. 5**)**.

**Table 3:** Multivariate logistic regression analysis of potential risk factors and lipids in CCVD subjects

Characteristic	Univariate		Model 1		Model 2	
	*P-Value*	OR (95% CI)	*P-Value*	OR (95% CI)	*P-Value*	OR (95% CI)
Male	0.004	1.841(1.217–2.785)	0.124	1.474(0.899–2.416)	0.419	1.290(0.695–2.394)
Age	0.003	1.027(1.009–1.045)	0.205	1.013(0.993–1.034)	0.719	0.995(0.969–1.021)
DM	0.004	1.871(1.222–2.867)	0.930	1.023(0.613–1.709)	0.356	1.813 (0.513–6.402)
AF	0.040	1.788(1.027–3.113)	0.258	1.415(0.775–2.582)	0.076	3.405(0.881–13.161)
IMT	0.000	5.756(2.287–14.489)	0.016	3.469(1.264–9.517)	0.019	4.361(1.271–14.964)
CPT	0.000	1.288(1.125–1.474)	0.154	1.126(0.957–1.324)	0.249	1.187(0.887–1.589)
Glucose	0.000	1.127(1.060–1.198)	0.050	1.074(1.000–1.153)	0.617	1.022(0.938–1.114)
TC	0.011	0.779(0.642–0.946)	0.138	0.849(0.685–1.054)	0.214	0.549(0.213–1.413)
HDL-C	0.003	0.433(0.247–0.761)	0.317	0.711(0.364–1.388)	0.363	0.545(0.147–2.016)
FFA	0.000	5.161(2.123–12.541)	0.001	5.660(2.123–15.091)	0.000	16.128(4.511–57.665)

Model 1: unadjusted

Model 2: all potential risk factors including age, BMI, gender, smoking, hypertension, DM, personal history of cardiovascular and cerebrovascular diseases, history of AF, lack of exercise, fatty liver, obstructive sleep apnoea, and CP were adjusted

*CCVD* Cardiovascular and cerebrovascular diseases, *OR* Odds ratio, *CI* Confidence interval, *DM* Diabetes mellitus, *AF* Atrial fibrillation, *IMT* Intima-media thickness, *CPT* Carotid plaque thickness, *TC* Total cholesterol, *HDL-C* High-density lipoprotein cholesterol, *FFA* Free fatty acid

## Discussion

The population in our study came from Changning District, one of the most prosperous communities among the 16 districts of Shanghai. In 2020, The GDP of Changning District was 156.117 billion yuan, and the resident population was 693,051. Residents of the Changning district were relatively affluent. In this study, the prevalence of MASLD was 38.1%, and the prevalence of DM was 21.1%. CCVD occurred in 12.6% of the patients. CP was present in 57.9% of all patients and 60.5% of MASLD patients. Although this data showed that atherosclerosis burden increased in the presence of MASLD, this association may be due to the fact that the samples in this study were from inpatients and outpatients of our hospital, which led to the high proportion of CCVD and CP. The high prevalence of DM may be the cause of the high prevalence of MASLD [27]. Our results also showed that older age, male, hypertension, history of AF, DM, and CP were independent risk factors for CCVD events, which was consistent with other studies [28–30]. However, there was no significant correlation between CCVD and the risk factors symbolizing overweight, which were MASLD and dyslipidemia, but it was positively correlated with hyperglycemia, TC, FFA, and negatively correlated with HDL.

We found common risk factors for CCVD events, fatty liver, and atherosclerosis, which included smoke, age, male, DM, and hypertension. Risk factors for CCVD and CP were similar. Many studies justify this association[31]. Our results may be due to MASLD and atherosclerosis having common pathogenesis, including endothelial dysfunction, systemic inflammation, hepatic insulin resistance, increased oxidative stress, and changes in lipid metabolism [32–34]. But the relationship between MASLD and atherosclerosis has been controversial. It is still no conclusion that if it is the association between MASLD and atherosclerosis reflects the underlying metabolic syndrome risk factor which further contributes to the much more severe atherosclerosis, or the MASLD leads to the development of atherosclerosis independently [35–37]. Our study did not find a direct relationship between MASLD and CCVD, but most patients with MASLD diagnosed by ultrasound have CP, although the correlation was weak (*P* = 0.016). In the carotid plaque group population of this study, there was no significant difference in the distribution of CPT and IMT between MASLD and non-MASLD patients. Interestingly, we found that in both CCVD and MASLD patient groups, carotid plaque thickness was mostly distributed in the CPT 2-4 mm group. This may be similar to plaque characteristics distribution in patients in the real world. Our multivariate logistic regression analysis was shown that CCVD was independently and consistently associated with higher IMT and FFA. Regarding the CPT, statistically significant relationships with CCVD were observed less frequently and were less robust. After adjustment, CPT was no significant relationship with CCVD. This may be due to the lack of information on plaque characteristics in this study, which included only the thickness of the plaque. We should probably pay more attention to the characteristics of plaques and use various imaging techniques to observe the vulnerability of the plaques, not just the degree of stenosis. In our previous study on carotid plaque assessment with contrast-enhanced ultrasound [38], we found that the contrast agent mainly infused from the surface to the interior of the plaque on contrast-enhanced ultrasound (CEUS), and increased serum FFA levels were indicative of vulnerable carotid plaques. We speculated the appearance of hypertension and FFA may cause the variation of the Contrast-enhanced perfusion patterns. That was closely related to the result of our study. Some previous studies also have shown serum FFA levels were closely related to lipid metabolism and MASLD [39]. MASLD may be related to widespread abnormal peri-organ or intra-organ fat (APIFat) deposition, such as epicardial adipose tissue, which may further contribute to cardiovascular risk [40]. MASLD is established as an integral component of the cardiometabolic-renal-liver complex. There is evidence that a two-way relationship between MASLD and several cardiometabolic risk factors, such as metabolic syndrome, hypertension, type 2 diabetes, central overweight, dyslipidemia, and chronic kidney disease [41]. It is reasonable to assume that the same pathways that produce MASLD may also lead to atherosclerosis. As these conditions develop, the liver may be involved as a target for metabolic abnormalities on the one hand, and an adverse metabolic burden on the other, leading to a vicious cycle. Thus, the development and evolution of MASLD may accelerate the formation of atherosclerosis [42, 43], but is not involved in the process of plaque damage and healing leading to increased plaque thickness.

Previous studies reported that the pathogenesis of MASLD can be partially explained by a metabolic component and partially explained by a genetic component [44, 45], the mechanisms of which are fundamentally different. The characteristic of metabolic components is a substrate surplus and increased rates of adipose tissue lipolysis and hepatic de novo lipogenesis [45]. A person with sarcopenia-linked MASLD, may develop central obesity MASLD over time, which may cause several other metabolic complications. Similarly, in most cases, patients with pre-diabetes will gradually develop into overt diabetes, and even into diabetes with cardiovascular or other system end-organ damage [46].

There were some limitations in our study. Firstly, this was a cross-section study, with all its inherent limitations. In particular, as the carotid plaque images were only obtained at a single time point, we could not determine how the carotid artery measurements and fatty livers change over time, nor can we prove a causal relationship. Although our study lacks follow-up data, we will continue to supplement it. Secondly, liver biopsy remains the gold standard for diagnosing MASLD and grading hepatic steatosis. However, the biopsy is costly, invasive, inappropriate for screening and follow up and it is not acceptable to most patients [47]. A meta-analysis showed that liver ultrasonography was an accurate, reliable tool to detect moderate to severe fatty liver, together with the relatively low cost and lack of radiation exposure. Though it had a low performance for the detection of mild steatosis, still supported the use of ultrasound as the imaging technique of choice for screening for fatty liver in clinical settings and population studies [48]. Conventional ultrasound is limited by its qualitative nature, operator dependency, and modest accuracy, but we make an analysis of the consistency between observers in the definition of MASLD, and it has a good overall inter-observer agreement. The images of CP were assessed by a ultrasound physician with 13 years of ultrasound experience, our results showed the maintenance of excellent intra-observer repeatability of CPT. Thirdly, all samples in our study were from symptomatic patients in outpatient and hospitalization in Changning District. Most of the samples had carotid plaques, which may be deviated from the real world. Finally, since all participants were from the Changning community in Shanghai, they could not be considered representative of the Chinese population. We will conduct a prospective multi-center project to verify our results in the future. And our multi-center project is in preparation which has passed the review of the Chinese Clinical Trial Registry (ChiCTR2000040163).

## Conclusions

According to our results, we recommend carotid ultrasound examination of the patients when FFA is increased, regardless of the presence of risk factors and MASLD. Contrast-enhanced ultrasound is necessary to assess the vulnerability of the plaque when CPT ≥ 2 mm. Timely treatment of vulnerable plaques may reduce the incidence of future CCVD events.

## Data Availability

The datasets used and/or analysed during the current study are available from the corresponding author on reasonable request.

## Abbreviations

MASLD: Metabolic dysfunction-associated steatotic liver disease
CCVD: Cardiovascular and cerebrovascular diseases
DM: Diabetes mellitus
HDI: Human development index
CP: Carotid plaque
CPT: Carotid plaque thickness
IMT: Intima-media thickness
CEUS: Contrast-enhanced ultrasound
AF: Atrial fibrillation
BMI: Body mass index
ICC: Intra-class correlation
FBG: Fasting blood glucose
TC: Total cholesterol
TG: Triglycerides
HDL-C: High-density lipoprotein cholesterol
LDL-C: Low-density lipoprotein cholesterol
Lp(a): Lipoprotein(a)
APOA: Apolipoprotein A
APOB: Apolipoprotein B
APOE: Apolipoprotein E
FFA: Free fatty acid
SDLDL-C: Small and dense low-density lipoprotein cholesterol
NHDL-C: Non-high-density lipoprotein cholesterol
OR: Odds ratio
